# Rapunzel syndrome: emergency laparoscopic management

**DOI:** 10.1093/jscr/rjag010

**Published:** 2026-01-30

**Authors:** Raul Yepez Adrianzen, Alejandro Irrazabal Ampuero, Angie Mariños Claudet

**Affiliations:** Emergency Surgery and Surgical Critical Care Service, Guillermo Almenara National Hospital, Lima 15018, Peru; Emergency Surgery and Surgical Critical Care Service, Guillermo Almenara National Hospital, Lima 15018, Peru; Emergency Surgery and Surgical Critical Care Service, Guillermo Almenara National Hospital, Lima 15018, Peru

**Keywords:** trichobezoar, Rapunzel syndrome, trichophagia, trichotillomania, bowel obstruction, psychiatric disorder

## Abstract

Rapunzel syndrome is a rare form of trichobezoar, typically associated with an underlying and often untreated psychiatric disorder such as trichotillomania. This case report describes a 24-year-old female patient previously diagnosed with trichotillomania and trichophagia, who presented with abdominal distension and vomiting due to a large gastric bezoar extending into the duodenum. Diagnostic evaluation included upper endoscopy and computed tomography, confirming complete upper gastrointestinal obstruction. Emergency laparoscopic management was successfully performed, allowing complete extraction of the trichobezoar, followed by gastric repair. Postoperative recovery was favorable, and psychiatric follow-up was initiated to address the underlying condition and reduce the risk of recurrence. Timely diagnosis enables successful minimally invasive surgical treatment and underscores the need for coordinated multidisciplinary management.

## Introduction

Rapunzel syndrome is a rare clinical entity characterized by the presence of a gastric trichobezoar that extends beyond the pylorus into the small intestine, causing obstruction, perforation, or gastrointestinal bleeding. First described in 1968 by Vaughan *et al*., and since then isolated cases have been reported [1]. Although Rapunzel syndrome itself is exceedingly rare with no reliable prevalence estimates, the underlying psychiatric condition—trichotillomania—has a reported prevalence ranging from 0.06% to 4% in the general population [2]. There are no clear reports of the incidence of this syndrome in Latin American countries due to limited mental health data. As of 2019, 120 cases had been reported in the medical literature. The condition is more frequent in females, with a peak age of onset between 5 and 23 years [3].

Trichobezoar formation is associated with trichophagia, which often coexists with trichotillomania. Repeated ingestion of hair, which is resistant to digestion and peristalsis, leads to progressive accumulation in the stomach and, in some cases, to the distal extension characteristic of the syndrome [4].

Patients typically present with abdominal pain, nausea, vomiting, weight loss, anemia, and a palpable abdominal mass. Due to its rarity and variable presentation, diagnosis is often delayed. Imaging studies such as computed tomography and upper gastrointestinal endoscopy are essential [5].

The treatment of choice is surgical resection of the bezoar, usually via laparotomy, although there are reports of successful laparoscopic approaches [6, 7]. Additionally, psychiatric evaluation and follow-up are indispensable to prevent recurrence [8]. We present the case of Rapunzel syndrome diagnosed and treated at the Institution, highlighting the clinical, diagnostic, and therapeutic findings, and discussing the importance of a multidisciplinary approach.

## Case report

Twenty four-year-old woman admitted to the emergency room with abdominal distension and vomiting. An upper endoscopy was performed, revealing a gastric bezoar. She had a prior diagnosis of trichotillomania since 2014 and reported persistent trichophagia for more than two years before admission, without receiving psychiatric follow-up.

Gastroenterology recommended a contrast-enhanced computed tomography (CT) scan and psychiatric consultation. Her complete blood count showed a white blood cell count of 29 560 cells/μl with 15% band neutrophils and a hemoglobin level of 9.2 g/dl.

The emergency surgery team recommended evaluation by Gastroenterology. Gastric Surgery Service recommended a medical board meeting within 72 h.

**Figure 1:**
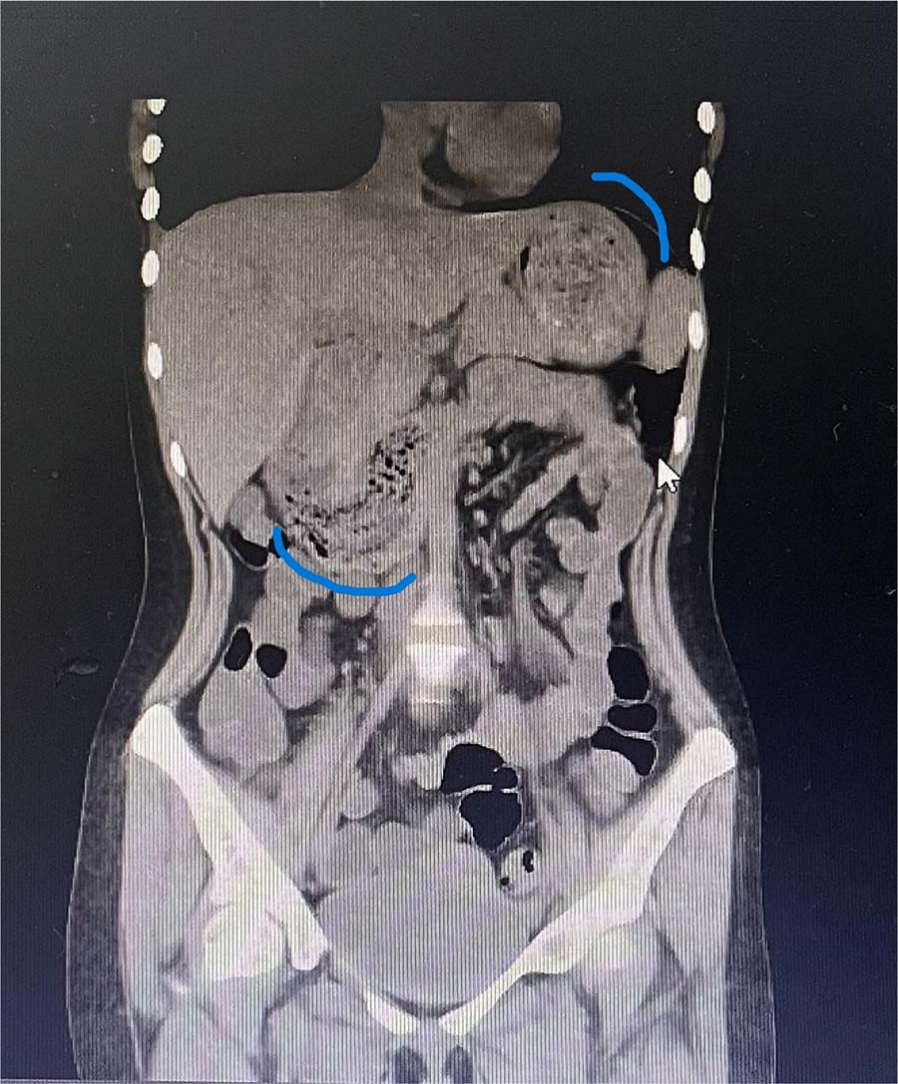
Sagittal CT image showing the foreign body occupying the gastric cavity and extending into the third and fourth portions of the duodenum.

**Figure 2:**
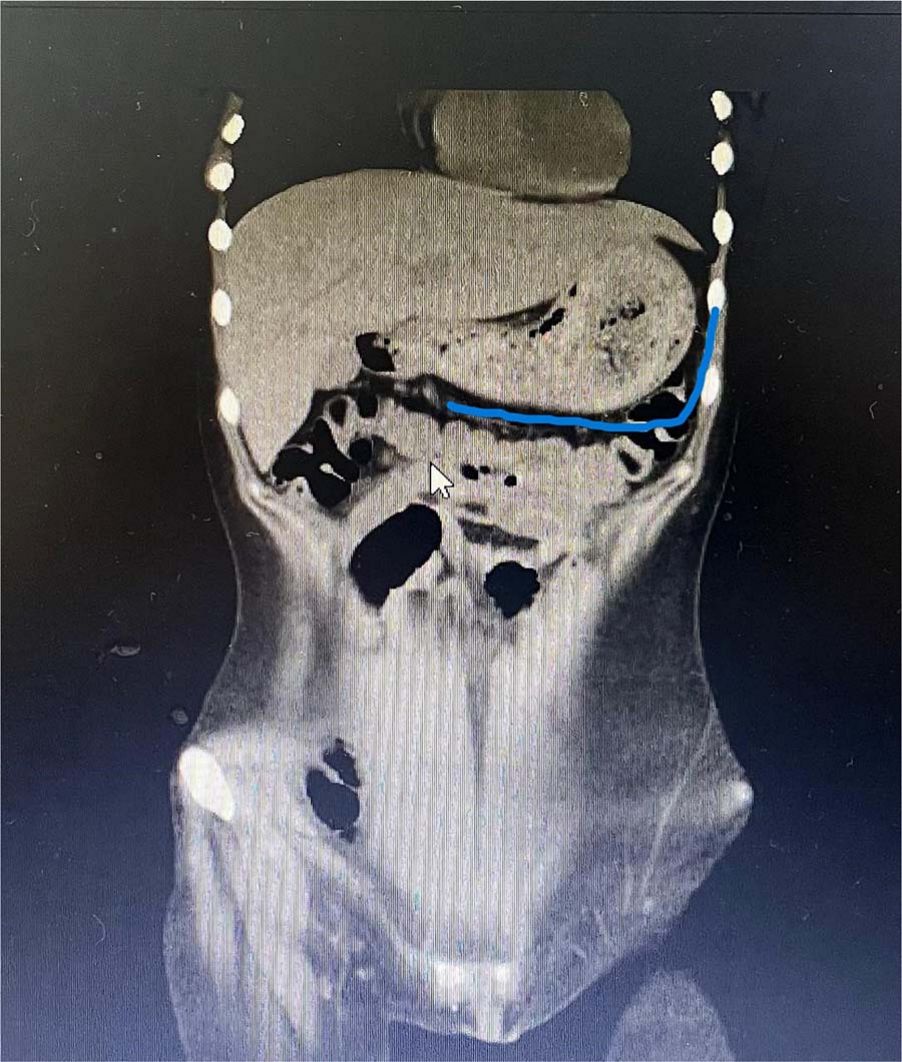
Sagittal CT image demonstrating the foreign body filling the entire gastric cavity.

**Figure 3:**
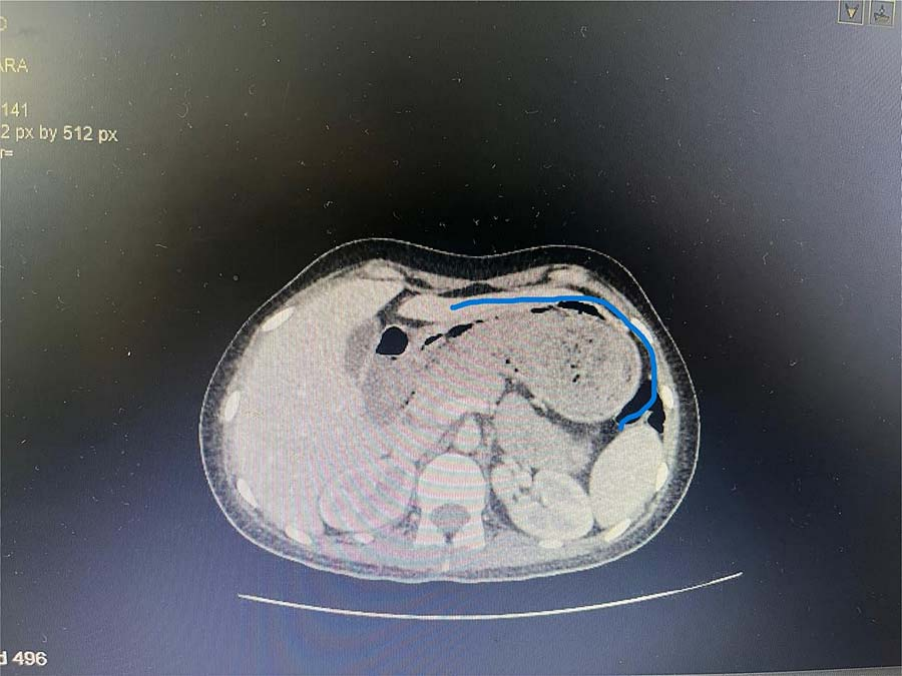
Axial CT image showing the foreign body completely occupying the gastric lumen.

**Figure 4:**
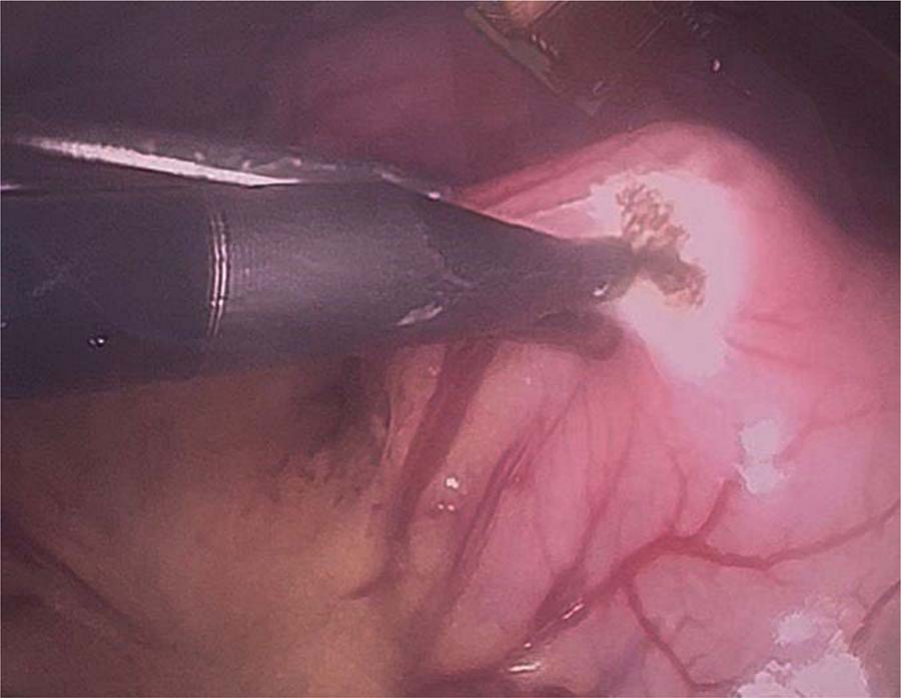
Initial gastrotomy beginning at the antral region and extending longitudinally toward the pylorus (without compromising it).

**Figure 5:**
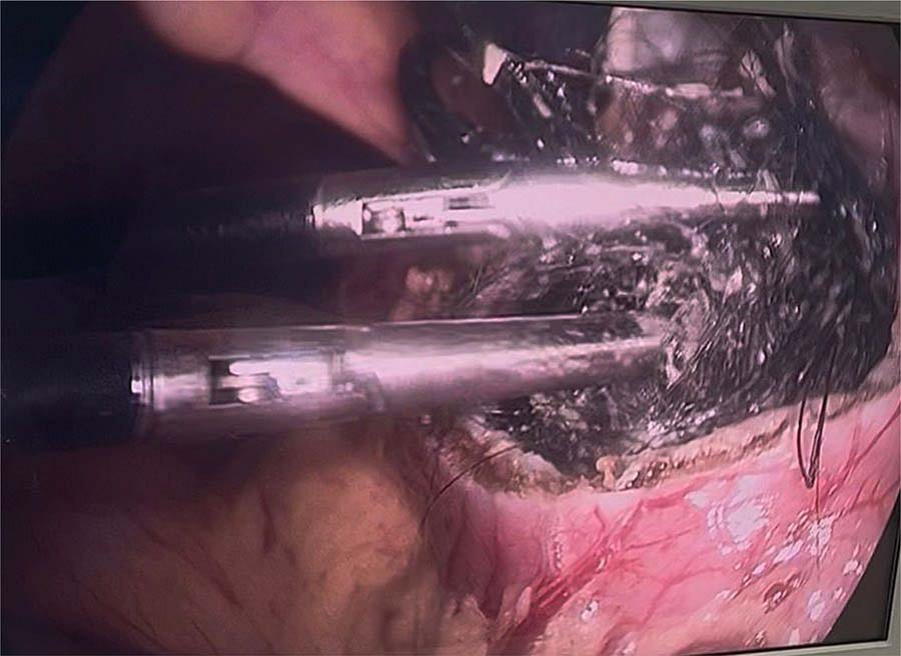
Identification of the bezoar after gastrotomy, revealing a large amount of contaminated trichobezoar material.

**Figure 6:**
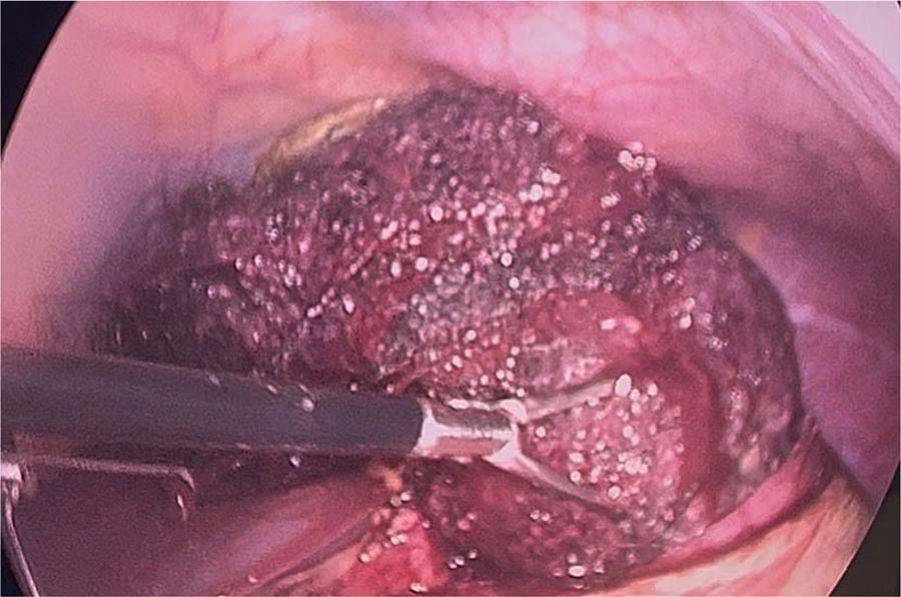
Bezoar extraction initiated from the duodenal region.

**Figure 7:**
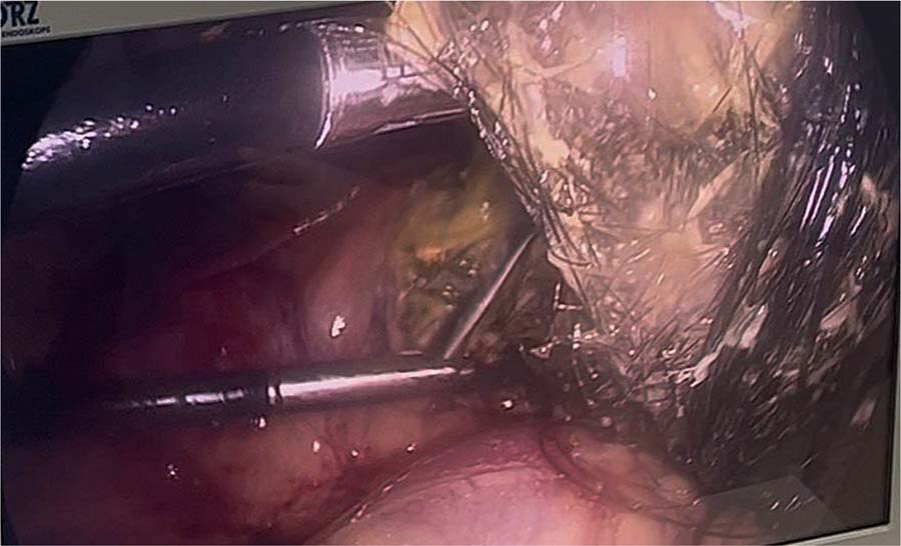
Final extraction of the bezoar from the gastric cavity and distal esophagus.

**Figure 8:**
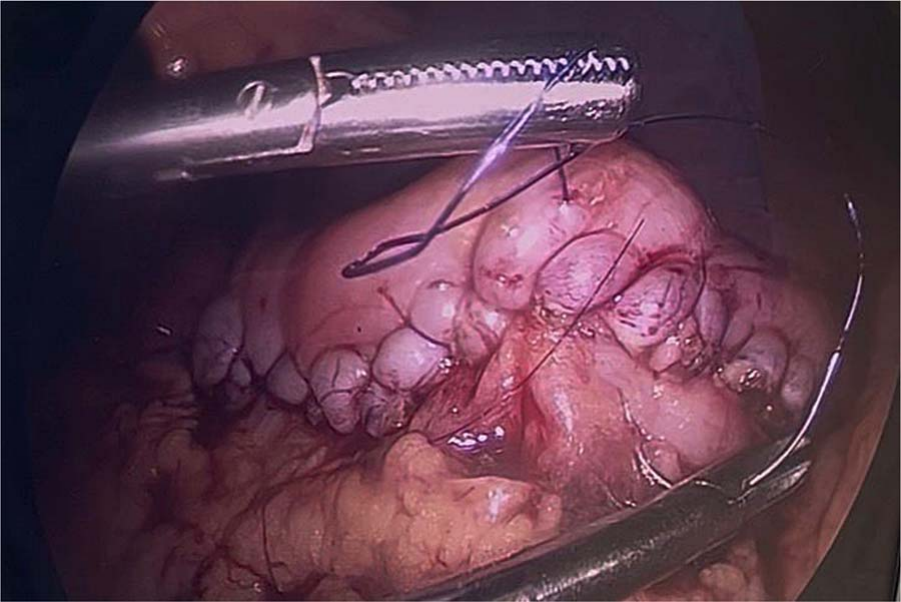
Final gastric closure using continuous single-layer suturing with 3–0 PDS on a round needle.

**Figure 9:**
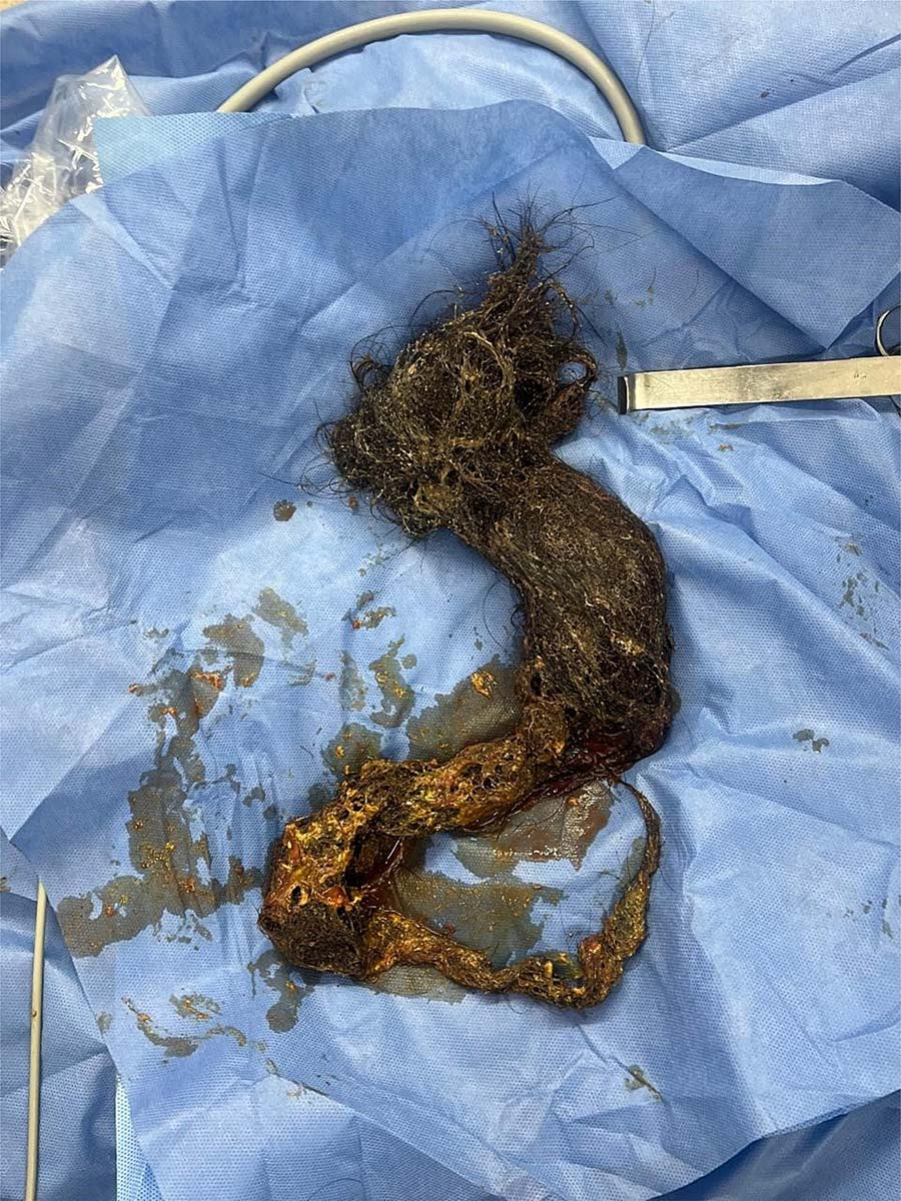
Surgical specimen of the bezoar.

**Figure 10:**
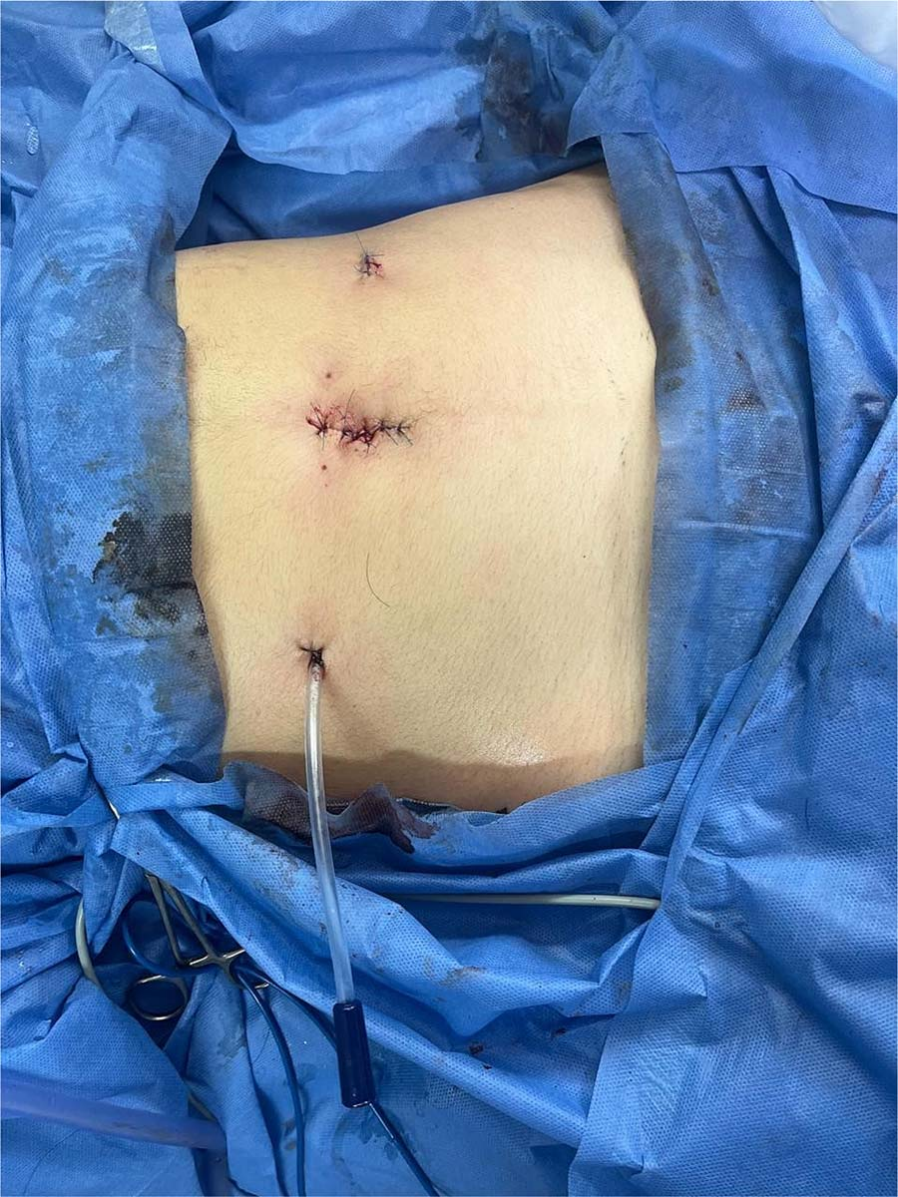
Final port placement at the end of the procedure, showing the tubular drain in situ.

Twelve hours later, she was evaluated by the new emergency surgery team, who found a complete upper obstruction with a foreign body occupying the lower esophagus, the entire stomach, and the entire duodenum (Figs 1–3). A diagnostic laparoscopy (Fig. 10) with gastrotomy was performed (Figs 4 and 5), including foreign body removal (Figs 6 and 7), gastric repair (Fig. 8), and placement of a tubular drain. The findings revealed a large foreign body containing a significant amount of malodorous hair, measuring 30 cm long and 12 cm wide (Fig. 9). Large gauze pads were placed around the stomach to prevent contamination, and the bezoar was removed first through the duodenal area, as this was the area of smallest diameter and least compression. The gastric and esophageal portions were then removed. The foreign body was extracted transumbilically through a small 6 cm incision, protecting the abdominal wall with an isolation device. Gastric repair was performed using a 3–0 continuous suture polydioxanone (PDS) in a single layer. The cavity was irrigated with 3 l of saline solution. The operation was 2 h. Postoperative management included a nasogastric tube on gravity drainage, intravenous piperacillin/tazobactam 4.5 g every 6 h, intravenous tramadol 100 mg every 8 h for analgesia, and intravenous dimenhydrinate 50 mg every 8 h. The patient progressed favorably, with oral intake initiated on postoperative day 4. The tubular drain was removed on day 5 with minimal serous output. Psychiatry discovered that the patient had been diagnosed with trichotillomania in 2014 but refused pharmacological treatment. The patient confirmed that she has suffered from trichophagia since the age of 14. Pharmacological treatment with psychotropic medications and psychological support was initiated. On day 7, the patient was on a soft diet, without a nasogastric tube, with a white blood cell count of 10 740 cells/μl and 0% band neutrophils. Treatment with clomipramine was initiated. She was discharged that day and evaluated 7 days later with no problems.

## Discussion

A 24-year-old patient who did not receive psychiatric treatment despite being diagnosed with trichotillomania 2 years prior. Thirty percent of people with trichotillomania have trichophagia, although only 1% of those with trichophagia develop a trichobezoar. A complete upper gastrointestinal obstruction is an emergency requiring emergency surgical management. It can be treated with upper endoscopy in cases of small masses or may require more invasive procedures via laparoscopy or even laparotomy. In this case, the delay in referring the patient to gastroenterology and, subsequently, the initial hesitation by the emergency surgery team led to a deterioration in the patient’s health [9, 10].

The possibility of laparoscopic surgery is evident and should be considered the gold standard surgical treatment, but it requires experienced personnel due to complications such as peritoneal contamination from an infected bezoar or potential dehiscence after gastric and/or duodenal repair. Laparoscopic removal of trichobezoars has advantages compared to laparotomy, including a better cosmetic outcome, fewer postoperative complications, and a shorter hospital stay. Several techniques for removing the bezoar laparoscopically have been described. Nirasawa *et al*. described the use of an 8-cm laparoscopic gastrotomy, placement of the bezoar in a plastic bag, and its subsequent extraction via a suprapubic minilaparotomy. However, this can lead to contamination of the surgical wound or the abdominal cavity [11].

Kanetaka *et al*. described a 1-cm gastric incision followed by fragmentation of the bezoar with laparoscopic scissors. However, fragmenting a bezoar is difficult [12].

Laparoscopic removal can be attempted provided certain conditions are met, such as the absence of complications, severity of the obstruction, and hemodynamic instability [13].

In our technique, we emphasize the strategic placement of gauze around the gastric incision to prevent the bezoar from contacting and contaminating the abdominal cavity. We also developed a method that enables removal of the bezoar without necessitating a large gastric incision, optimizing the advantages of a minimally invasive approach.

Given the potential for contamination in cases involving infected bezoars or gastric spillage, appropriate antimicrobial coverage is essential to reduce postoperative infectious complications. According to international World Society of Emergency Surgery guidelines, piperacillin–tazobactam is considered a first-line agent for contaminated acute abdominal emergencies [14].

The recurrence rate, according to Vásquez-Ciriaco *et al*. can reach 16%; therefore, the patient should not be discharged without psychiatric management [15].

Mental health is undervalued worldwide and can lead to these types of emergency surgical complications, even though they are not frequent. Emergency surgeons must have the ability to resolve these types of obstructive cases.
